# Workshop on Use of Intravenous Immunoglobulin in Hand, Foot and Mouth Disease in Southeast Asia

**DOI:** 10.3201/eid2101.140992

**Published:** 2015-01

**Authors:** Sokkosal Chea, Yi-bing Cheng, Kulkanya Chokephaibulkit, Tawee Chotpitayasunondh, H. Rogier van Doorn, Zen Hafy, Surinda Kawichai, Ching-Chuan Liu, Nguyen Tran Nam, Mong How Ooi, Marcel Wolbers, Mei Zeng

**Affiliations:** Angkor Hospital for Children, Siem Reap, Cambodia (S. Chea); Zhenzhou Children’s Hospital, Henan, China (Y. Cheng);; Siriraj Hospital, Mahidol University, Bangkok, Thailand (K. Chokephaibulkit);; Queen Sirikit National Institute of Child Health, Bangkok (T. Chotpitayasunondh);; Oxford University Clinical Research Unit, Ho Chi Minh City, Vietnam (H.R. van Doorn, M. Wolbers);; Family Health International 360, Bangkok (Z. Hafy, S. Kawichai);; National Cheng Kung University, Tainan, Taiwan (C.-C. Liu);; Children’s Hospital 2, Ho Chi Minh City (N.T. Nam);; Sarawak General Hospital, Kuching, Sarawak, Malaysia (M.H. Ooi);; Fudan University Children’s Hospital, Shanghai, China (M. Zeng)

**Keywords:** enterovirus, enterovirus 71, hand foot and mouth disease, intravenous immunoglobulin, Southeast Asia, workshop, IVIg

## Abstract

The South East Asia Infectious Disease Clinical Research Network convened subject matter experts at a workshop to make consensus recommendations for study design of a clinical trial for use of intravenous immunoglobulin (IVIg) in severe hand, foot and mouth disease (HFMD). HFMD is a highly contagious emerging infection among children in the region, a small proportion of whom develop neurologic and cardiopulmonary complications with high case-fatality rates. The use of IVIg for treatment of severe disease is widespread and a part of local, national, and international guidelines, but no clinical evidence warrants the use of this drug, which is expensive and has potentially serious side effects. During a 2-day workshop in March 2014, a group of HFMD experts reviewed the current evidence related to use of IVIg in HFMD and discussed potential study design, feasibility, inclusion and exclusion criteria, sample size, primary and secondary endpoints, and subsidiary studies for a randomized, placebo-controlled trial.

## Abstract 2

A randomized, placebo-controlled clinical trial is needed to determine efficacy and safety of this treatment method.

Hand, foot and mouth disease (HFMD) is typically a benign, self-limiting illness among young children and infants that is characterized by fever; ulcerating vesicles in the mouth; and lesions, usually maculo- or papulovesicular, on hands, feet, and buttocks. Lesions usually occur on the palms and soles. Outbreaks occur worldwide and are often associated with daycare centers, kindergartens, and elementary schools (*1*,*2*). HFMD is caused by enteroviruses belonging to the species *Enterovirus A* (serotypes coxsackievirus A 2–8, 10, 12, 14, and 16 and enterovirus [EV] 71, 76, and 89–92) and, rarely, *Enterovirus B* (*3*). *Enterovirus A* and *Enterovirus B* species can also cause herpangina, which may overlap clinically with HFMD. Since the late 1990s, EV71 has been associated with large outbreaks in the Asia-Pacific region. Young children (median age <2 years) are affected, and some experience severe illness that may lead to neurologic and cardiopulmonary complications and even death.

## Enteroviruses

Humans are the only known host for enteroviruses, which are nonenveloped viruses and, therefore, highly resistant to environmental conditions but also to mild disinfectants such as 70% ethanol and ethanol-based hand rubs. Transmission of enteroviruses is oro-fecal, but HFMD may also be transmitted by the respiratory route; enteroviruses have been shown to replicate in the tonsils (*4*) and can be detected in respiratory secretions (*5*). Enteroviruses are acid-resistant and can pass through the stomach after ingestion, after which they replicate in the gut and spread through the reticuloendothelial system (*1*).

### EV71

EV71 is thought to have evolved from coxsackievirus A16 around 1940 (*6*) and has diverged into 3 lineages: A, B and C. Lineages B and C are further divided into the B1–5 and C1–5 sublineages. Although genetically different, all lineages and sublineages represent 1 serotype, EV71. 

EV71 was initially detected in a child with encephalitis in 1969 in California and was then retrospectively detected from an isolated case from 1963 in the Netherlands (*7*,*8*). Large outbreaks with high death rates resulting from EV71-associated encephalitis have been reported from Bulgaria (1975), Hungary (1978), Sweden (1973), and Australia (1972) (*9*–*13*). A small number of patients in the Bulgaria outbreak showed clinical signs and symptoms of HFMD (*13*), but the first clear associations between HFMD and EV71 were seen during outbreaks in Japan in 1973 and 1978 (*14*,*15*). C5, C4, and B5 have been the dominant EV71 sublineages in outbreaks occurring during recent years in Southeast Asia (*16*,*17*).

### Outbreaks of EV71 in Asia

Currently, EV71-associated HFMD is endemic in large parts of Southeast Asia. Large outbreaks have occurred in Taiwan in 1998 (≈1,500,000 cases and 78 deaths) (*18*); in China in 2008 and 2009 (490,000 and 1,150,000 cases and 127 and 353 deaths, respectively) (*19*); in Vietnam in 2011–2012 (260,000 cases and 211 deaths) (*17*); and in Cambodia in 2012 (unknown total number of cases and >54 deaths) (*20*). In the Taiwan outbreak, EV71 was isolated in 49% of patients with uncomplicated infection, in 75% of hospitalized patients, and in 92% of the 78 patients who died; this evidence suggests that EV71 is strongly associated with severe illness and death (*18*). A 2–3-year EV71 outbreak pattern has been described in Japan, Taiwan, Malaysia, and provinces of China (*21*), with seasons of less severe HFMD caused by the other enterovirus A types between the EV71 peak years. This pattern is assumed to relate to the presence of birth cohorts of children who have not been exposed to the virus of a size that is large enough to sustain human-to-human transmission. Coxsackievirus A6 and A10 have been dominant in the region recently (*22*,*23*).

### Neuroinvasion and Severe Illness

EV71-associated HFMD is distinct from non-EV71 HFMD in clinical manifestations that are more severe in a minority of patients. Data from mouse studies suggest that, after initial replication in the gut and the reticuloendothelial system, EV71 can invade the central nervous system (CNS) through retrograde axonal spread along cranial or peripheral nerves (*24*).

EV71 can cause aseptic meningitis, acute flaccid paralysis, encephalitis, and other rare manifestations. Myoclonic jerks are seen more often in cases of EV71 infection than during infections with other enteroviruses and could be an early indicator of neurologic involvement, particularly in the brainstem. Brainstem encephalitis with autonomic dysregulation and associated neurogenic pulmonary edema has been the hallmark of the severe end of the spectrum of EV71 CNS infection in Asia since the late 1990s. After 3–5 days of prodromal HFMD and high fevers, acute and rapidly progressing cardiorespiratory failure develops in children, beginning with symptoms of shock and pulmonary edema or hemorrhages (*25*). Postmortem studies of human EV71 infections have shown limited viral infection of neuronal cells of the central parts of the brain and inflammation in the spinal cord, medulla, pontine tegmentum, midbrain, hypothalamus, and subthalamic and dentate nuclei (*4*). The exact mechanism for autonomic dysfunction and pulmonary edema in EV71 encephalitis is unclear, but neurogenic mechanisms secondary to brainstem invasion and inflammation, especially of the vagus nucleus, seem to be important (*24*).

## Background of the Workshop

On March 4–5, 2014, in Bangkok, Thailand, the South East Asia Infectious Disease Clinical Research Network (SEAICRN) convened subject matter experts at a workshop to make consensus recommendations for study design of a clinical trial for use of intravenous immunoglobulin (IVIg) in severe HFMD. During earlier teleconferences, SEAICRN members had discussed the widespread use of IVIg in severe HFMD, as had been recommended by the World Health Organization (WHO) (*26*) and local ministries of health, despite the lack of evidence for its benefit to patients. Members discussed the need for a study of the efficacy of IVIg to prevent progression of disease severity and safety concerns surrounding use of this treatment and agreed that a randomized, placebo-controlled trial is needed. The following potential hurdles for a multicenter multinational trial were identified:

1. National and local guidelines for management of severe HFMD use different grading systems and have different indications for various interventions, including IVIg.
2. Different brands of IVIg are used in the various countries in the region, and there are no parameters of efficacy on which a rational choice could be based because the mechanism of action of IVIg is unknown.
3. Because IVIg is part of some national guidelines, is considered standard of care, and/or is covered by national health insurance schemes, some countries may not be able to participate in a placebo-controlled trial because it would contravene standard-of-care.

As alternatives, a matched control design or a purely observational study was discussed. The latter potentially could be run as an additional arm to the trial, to allow participation of centers in countries where a trial would not be feasible because of ethical constraints; in this instance, clinical data and samples would be collected using the same platform as a placebo-controlled trial, enabling pooling and simultaneous analysis of data.

Before the workshop, participants were asked to submit (translations of) their national or local guidelines. These were summarized (Technical Appendix), and main similarities and differences were highlighted and discussed during the workshop and follow-up teleconferences. Participants were also asked to submit details regarding the brand and dosage of IVIg used in their country/center. The purpose of the workshop was to discuss these differences and to reach consensus on trial design. The meeting started with short introductions and presentations from the participants about their centers, numbers of cases, management guidelines, and IVIg indications and use in their centers and in each country generally.

## Clinical Grading and Management of HFMD

Most HFMD management guidelines, including the 2011 WHO guidelines (*26*), use a clinical grading system that measures the severity and stage of illness. Although there are several large differences, all are largely similar in the sense that they all describe 4 stages (in order of increasing severity): 1) uncomplicated HFMD/herpangina, 2) with CNS involvement, 3) with autonomic dysregulation, and 4) with cardiopulmonary failure. Supportive measures and interventions are dictated by the grade of severity.

Clinical management of HFMD is largely supportive in nature; no specific antiviral drug treatments are recognized. Children with uncomplicated HFMD are best treated at home. Children who have warning signs or frank signs and symptoms of CNS involvement should be hospitalized and closely monitored for further complications that may require intensive care and supportive measures. Severity and duration of fever are independent risk factors for CNS involvement. Vomiting, lethargy, agitation, and irritability have also been shown to be associated with CNS involvement (*27*,*28*). More specific neurologic signs, such as myoclonic jerks (usually observed during the early stage of sleep but also seen in severe cases when patients are awake), limb weakness, truncal ataxia, and “wandering eyes” (rotary eye movement without fixation), are commonly observed in children in the early stage of severe disease (*29*). Other warning signs are irregular breathing pattern, dyspnea (labored breathing effort), tachypnea (increased breath rate), and mottled skin.

Although exact indications differ among countries in the region, IVIg is usually given to prevent further progression of illness in children with severe HFMD in the early stages of CNS involvement or autonomic dysregulation. The Technical Appendix highlights the relevant features of studied guidelines from the region.

## Use of Normal IVIg

Normal or polyclonal (as opposed to hyperimmune) IVIg is used for 3 purposes in the prevention and treatment of infectious diseases:

1. Supplementation therapy for immunocompromised patients with agammaglobulinaemia, severe combined or common variable immune disorders.
2. Passive immunization after measles or hepatitis A or B exposure (*30*–*32*)
3. As a panacea to treat infectious diseases for which there are no or insufficient antimicrobial drugs available. With the exceptions of Kawasaki disease, which is thought to be an infectious disease; Guillain-Barré syndrome, which is a postinfectious complication of an infectious disease; and some toxin-mediated infectious syndromes (*33*–*35*), little evidence exists for the benefit of IVIg treatment in infectious disease (*36*–*38*). This treatment method is used primarily when treatment alternatives are lacking.

IVIg use for HFMD started during large epidemics in which young children were dying and no antimicrobial drugs were available (*18*,*39*). Historic evidence for benefit of IVIg in enteroviral illnesses came from its use in infants with agammaglobulinemia and children with chronic enteroviral meningitis. In these cases, the drug was given as supplementation therapy and to suppress viral replication through neutralization by enteroviral antibodies. Patients often relapsed, however, when treatment was stopped and needed several courses of IVIg, intrathecal infusion, or chronic treatment (*40*–*42*). Some in vitro benefit (higher neutralizing titers, lower viral loads) was observed in neonates with enteroviral sepsis, but no differences in outcome were found (*43*,*44*)

In vitro data from HFMD patients showing a decrease of chemokine and cytokine titers and suggesting an immunomodulatory effect of IVIg are limited (*45*). IVIg may have a direct antiviral effect through neutralizing activity of enteroviral antibodies. However, many patients already produce anti-enteroviral IgM at the start of illness, and a lack of neutralizing antibodies is not expected to be the cause of the severe phenotype of disease that will develop in only a small proportion of children.

The use of IVIg in HFMD has yet to be supported by evidence from well-designed observational studies and randomized clinical trials. Some anecdotal experience in Asia indicates that IVIg, if administered early, could halt disease progression to autonomic nervous system involvement and subsequently to often fatal pulmonary edema. Comparisons with historical controls showed a benefit of the use of IVIg, but this comparison came in the context of implementation of stage-based management that included more than IVIg alone (*46*,*47*). An observational study from Malaysia showed that, among 224 children that had severe CNS complications, 204 (95%) of 215 children who survived had timely hospital admission and IVIg treatment compared with just 1 (11%) of 9 children who died (*28*,*48*). However, this uncontrolled study had a time-dependent bias; most patients who did not receive IVIg were extremely ill and died quickly, before they had the opportunity to receive IVIg.

Nevertheless, the use of IVIg has been adopted and recommended by several national and international guideline committees, including WHO (*26*), and is now part of standard of care for complicated HFMD in several countries and included in national insurance programs. However, the benefits and severe adverse events are not being studied systematically.

IVIg is not without risk. Although processing and purification have been optimized, the use of human blood products comes with myriad potentially severe side effects (*49*,*50*), including (fatal) anaphylaxis, which, although rare, typically occurs in immunocompetent patients and was also observed in 2 children in Vietnam (N.T. Nam, pers. comm.). In addition, a large infusion volume is required, and often these children are already experiencing hypertension and tachycardia; usual amounts are 2 dosages of 20 mL per kilogram of body weight. IVIg is also very expensive (in Vietnam, ≈USD80/g). As stated in the 2011 WHO guidelines, before use of IVIg can be recommended, randomized, preferably double-blind, controlled trials are required (*26*).

The advised total dosage of IVIg for most indications is 2 g/kg body weight. For HFMD, IVIg is usually given in 2 doses of 1 g/kg (total dosage 2 g/kg). The first dose is instilled over 8–12 hours; a second dose is given 24 hours after the start of the first dose, at the discretion of the treating physician. A similar dosing schedule would be used for the proposed clinical trial. Some doctors give higher doses, such as 3 or 4 doses of 1 g/kg); others have reported that a lower dose, such 0.5 or 1 g/kg, has shown effectiveness.

Over the past few years, 14 different preparations of IVIg from companies in Australia, Asia, Europe, and North America had been or were being used in the different centers reporting for the workshop. The attendees agreed that 1 brand of IVIg, produced in large batches of stable quality, would need to be used across all participating centers during the entire proposed trial. This preparation would need to be collected, purified, and checked for pathogens according to international safety guidelines. Although the mechanism of action of IVIg in HFMD is unknown, EV71 titers should be checked to ensure antiviral activity.

## Trial Feasibility

Workshop attendees agreed that a randomized, double-blind, placebo-controlled trial of IVIg for severe HFMD was necessary but would not be feasible in all countries because of national guidelines that dictate the use of IVIg and national insurance coverage for IVIg in some countries. Attendees from Taiwan, Thailand, and Malaysia thought trials would be unlikely to be run in those countries because of public awareness of and long experience with use of IVIg. Thailand, in addition, does not see many severe cases that would be eligible. In mainland China and Taiwan, IVIg is recommended by national guidelines, and national insurance covers IVIg in large parts of the country. In Cambodia, IVIg is not available for use. In Vietnam, although the national guidelines dictate IVIg use and national insurance covers all treatment in children <6 years of age, a similar study design has been discussed, and the Department of Health and the 2 tertiary pediatric referral hospitals in Ho Chi Minh City support a randomized, placebo-controlled trial.

## Trial Design

Through structured discussions, a number of suggestions for the design of a randomized, placebo-controlled trial of IVIg in the treatment of HFMD were made. These suggestions are described below.

### Inclusion and Exclusion Criteria

Numerous different grading and staging systems exist across the region. For clarity, it is therefore better to avoid the use of grades of severity or stages of diseases and instead use descriptive inclusion criteria. Inclusion criteria for patients to be randomized to receive IVIg or placebo should be in line with accepted clinical indications for IVIg, including those described in the 2011 WHO clinical management guidelines (*26*). IVIg currently is given to patients with the intention to slow down or prevent further progression of disease to a more severe stage. Two classes of patients with a clinical diagnosis of HFMD or herpangina would be eligible for randomization: those with signs and symptoms of neurologic involvement, and those without signs and symptoms of neurologic involvement but with signs and symptoms of imminent autonomic dysfunction. Patients who have already progressed to a more severe stage of disease would be excluded from the study (Technical Appendix).

### Primary Clinical Endpoints

Further progression of disease to a defined next level of severity will be the primary efficacy endpoint of the study. This endpoint is described as advanced-stage HFMD with frank autonomic dysregulation or cardiopulmonary failure (Technical Appendix). Patients will be unblinded immediately, even if the first study dosage is not completely instilled, when they reach the primary endpoint and will receive IVIg at the discretion of the treating physician. Adverse events and serious adverse events will be the primary safety endpoints, particularly those potentially related to either IVIg (fevers, chills, headache, aseptic meningitis, anaphylaxis) or fluid overload (edema and signs of congestive heart failure). Secondary endpoints and subsidiary studies are described in the Technical Appendix.

### Sample Size Justification

The exact proportions of patients receiving IVIg and not receiving IVIg who progress to a more severe stage of disease is not known. Sample size calculations used detailed data from >10,000 patients (including severity grade at admission, highest severity grade during hospitalization, and IVIg timing) collected at Children’s Hospital 2, Ho Chi Minh City, Vietnam, during April 2011–December 2012. The estimated rate of progression to more severe disease of children eligible to receive IVIg treatment was 17%–36% in Vietnam. 

Because establishing efficacy of IVIg is the primary research aim, a 1:1 randomized superiority trial would be the design of choice. However, because there are countries where IVIg is standard of care, establishing noninferiority of placebo might also be desirable. Sample size requirements for both scenarios with different absolute risk increases are displayed in Tables 1 and 2. We assumed that 17% was the most realistic estimate of progression in the IVIg group because this was the estimate obtained after excluding patients who received IVIg at a time when they had already reached a more severe stage of disease, similar to our exclusion criteria/primary clinical endpoint. (That is, we assumed illness in these children progressed too quickly to receive IVIg timely, so they would also meet exclusion criteria before being enrolled into the proposed trial.) The required total sample sizes for demonstrating superiority of IVIg (assuming the percentage with IVIg progression is 17% and ensuring ability to show the difference with placebo if the progression rate is 5% higher on placebo) at the 2-sided 5% significance level would be 1,970 or 2,636 participants for a desired power of 80% or 90%, respectively. A sample size of 2,636 would also have 80% power to detect an increase in the risk of progression from 17% to 23%, 24%, and 27% for subgroups of 60%, 40%, and 20% of the total population, respectively (e.g., age groups, PCR-confirmed EV71, or individual sites)

**Table 1:** Total required sample size for a 1:1 randomized trial designed to demonstrate superiority of IVIg compared with placebo at the 2-sided 5% significance level with 90% power*

Absolute risk of progression, IVIg group	Absolute risk increase for placebo group compared with IVIg			
	+5%	+7.5%	+10%	+15%
15%	2,424	1,136	670	322
20%	2,928	1,350	784	370

*Sample sizes are total sample sizes (i.e., for both groups combined) but do not account for loss-to-follow-up or increases in sample size that would be required if formal interim analyses with stopping boundaries are planned. IVIg, intravenous immunoglobulin.

**Table 2:** Total required sample size for a 1:1 randomized trial designed to demonstrate noninferiority of placebo compared with IVIg at the 1-sided 2.5% significance level with 90% power*

Absolute risk of progression†	Noninferiority margin Δ‡			
	5%	7.5%	10%	15%
15%	2,144	954	536	240
20%	2,690	1,196	674	300

*Sample sizes are total sample sizes (i.e., for both groups combined) but do not account for loss-to-follow-up or increases in sample size that would be required if formal interim analyses with stopping boundaries are planned. IVIg, intravenous immunoglobulin. †Assumed to be identical for both groups. ‡The noninferiority margin Δ is the absolute risk increase in the placebo arm to be exclude with the trial. That is, if the assumptions are correct, the sample sizes above guarantees that we can reject the hypothesis that placebo increases the absolute risk by +Δ (or more) at the 1-sided 2.5% significance level with 90% power. (Or, equivalently, the sample sizes above guarantee a probability of 90% that the 2-sided 95% CI for the absolute risk difference excludes an excess risk of +Δ, at worst, in the placebo arm.)

## Future Plans

A full protocol for a multinational, multicenter, randomized, placebo-controlled trial will be developed by using the recommendations from this workshop. Vietnam, Thailand, and Cambodia are the most likely sites for the trial to be able to enroll participants, and representatives of sites in these countries will be approached to request participation. Pharmaceutical companies that distribute IVIg in the region will also be contacted regarding their products and possibilities of placebo production. The protocol will subsequently be submitted to funding bodies for consideration.

## Conclusions

HFMD is a serious public health threat and burden to health care systems across Southeast Asia and associated regions. In the absence of region-wide vaccination coverage, systematic study of severe HFMD is needed, including proper assessment of the current interventions in use. IVIg is one of the most commonly used interventions but is expensive and has potentially severe side effects, so its use should be a primary subject of these assessments. During a 2-day workshop, guidelines and clinical management in different countries were discussed, and suggestions for the design of a randomized, double-blind, placebo-controlled trial were made. These suggestions will serve as the foundation for protocol development and further funding applications for a trial of IVIg use for treatment of severe HFMD.

Technical Appendix International and national guidelines for management of hand, foot and mouth disease (HFMD) and inclusion criteria, definitions, and primary endpoints for proposed trial of intravenous immunoglobulin (IVIg) in treatment of HFMD.
