# Supplementary material for: Workshop on Use of Intravenous Immunoglobulin in Hand, Foot and Mouth Disease in Southeast Asia
Source: Emerg Infect Dis. 2015 Jan;21(1):e140992. doi: 10.3201/eid2101.140992 (PMC4285270; doi:10.3201/eid2101.140992)
Supplement: Technical Appendix — International and national guidelines for management of hand, foot and mouth disease (HFMD) and inclusion criteria, definitions, and primary endpoints for proposed trial of intravenous immunoglobulin (IVIg) in treatment of HFMD. [file 14-0992-Techapp-s1.pdf]

Article DOI: http://dx.doi.org/10.3201/eid2101.140992

Workshop on Use of Intravenous Immunoglobulin in Hand, Foot and Mouth Disease in Southeast Asia

Technical Appendix

International and National Guidelines for Management of Hand, Foot and Mouth Disease (HFMD)

World Health Organization (WHO): The management algorithm of the 2011 WHO guidelines describe 4 grades of severity: uncomplicated HFMD, HFMD with central nervous system [CNS] complications, HFMD with autonomic nervous system [ANS] dysregulation, and HFMD with cardiopulmonary failure. Based on anecdotal experience that intravenous immunoglobulin (IVIg) may halt progression, if administered early, to ANS dysregulation, and subsequently to devastating pulmonary edema (*46*,*48*; references listed with main article text). IVIg is recommended for patients with encephalitis and flaccid paralysis, may be considered in brainstem encephalitis. IVIg is also recommended when there are ANS dysregulations and may be considered when there is cardiopulmonary failure and IVIg hasn't been given yet. IVIg is not indicated for patients with aseptic meningitis as these usually have a good prognosis (*26*).

Thailand: Thailand uses the WHO guidelines.

Cambodia: Unofficial guidelines used in Cambodia are based on a Taiwanese grading system (*51*). IVIg is recommended for stage II disease (aseptic meningitis/encephalitis/encephalomyelitis/polio-like syndrome). However, IVIg is currently not available for use in Cambodia.

Taiwan: The guidelines from Taiwan CDC 2011 describe 5 phases of disease (Phase 1: rash, Phase 2: neural system involvement, Phase 3: pre cardiopulmonary failure, Phase 4: cardiopulmonary failure, Phase 5: recovery). IVIg is not recommended for common phase 2 cases, but those with encephalitis or high fever and signs of pre-sepsis. For phase 3 cases, IVIg

can be considered in patients with drowsiness and frequent myoclonic jerks, or acute paralysis, respiratory rate >30–40 times/minutes at rest, heart rate >140–150 and signs of peripheral perfusion insufficiency such as sweating, cold extremities, or skin color change.

Malaysia: In Malaysia there are no national guidelines. The Sibu hospital in Sarawak uses a clinical management protocol that describes a staged approach similar to the stages used in the Cambodia and Taiwan (*51*), with encephalomyelitis as stage 2 and autonomic and cardiopulmonary dysfunction as stage 3. IVIg use is described for stage 2 and 3 but only after lumbar puncture (showing pleiocytosis) and consultation with a pediatrician, and is said to be less effective in stage 3. After 2011 the WHO guidelines have been adopted.

China: The 2011 consensus document from China CDC indication describes the following indications for IVIg: symptoms of hand-food-mouth disease or herpangina, or un-typical symptoms with consistent exposure history of enterovirus infection, and one of the following:

• myoclonic jerks AND tachycardia (heart rate >150/minute)

• acute paralysis

• acute encephalitis with brainstem involvement: ataxia, cross hemiplegia, or specific cranial nerve lession

• brainstem dysautonomia

• respiratory failure, acute pulmonary edema, pulmonary hemorrhage, ARDS

• heart failure

• sepsis syndrome

IVIg is not recommended for children >5 years of age, children with multiple organ failure and children with only meningitis but without encephalitis or paralysis, or patients with encephalitis not caused by enterovirus. Many patients will receive IV ribavirin or traditional Chinese medicine.

Vietnam: Vietnam uses a complicated grading system, bases on the system from Taiwan (*51*), but changed considerably during the large 2011–2012 outbreak (*17*). Grade 2 disease (CNS involvement) is split up in Grade 2a in which children have either a history of sporadic

myoclonus (not seen by doctor), prolonged or high fever or vomiting. In Grade 2b disease there are 2 groups. Group 1 has either myoclonus observed by doctor, a history of frequent myoclonus, lethargy or tachycardia >130 (afebrile and at rest). Group 2 has either ataxia, nystagmus, limb weakness (<4/5), cranial nerve paralysis, fever of >39°C (unresponsive to antipyretics) or tachycardia >150 (afebrile and at rest). Grade 3 is frank ANS dysregulation and Grade 4 is cardiopulmonary failure. Patients at grade 2a and b receive Phenobarbital to suppress myoclonus. IVIg is indicated for grade 2b group 2 and for grade 3, for grade 2b group 1 if signs do not improve after 6–12 hours with oral or intravenous Phenobarbital, and for grade 4 only after shock recovery (when average blood pressure ≥50 mm Hg).

Inclusion Criteria for Trial of IVIg in Treatment of HFMD

Inclusion criteria:

- Younger than 12 years
- Within 96 hours of onset of illness
- HFMD or herpangina or EV71 positive CSF PCR

AND

- meeting the criteria for either group A or B

Group A:

- Having any focal neurologic signs (other than aseptic meningitis or simple febrile seizures alone), defined as:
    - Alteration of consciousness (lethargic, irritable, drowsy, GCS <14) or other neurologic signs as:
    - Myoclonic jerks
    - Cerebellar signs as tremor/ataxia/nystagmus
    - Limb weakness
    - Acute flaccid paralysis
    - Cerebral nerve palsy

    - Wandering eyes
    - Other focal neurologic deficits

OR

- CSF abnormalities (pleiocytosis in the absence of red blood cells)

OR

- CT/MRI abnormalities suggestive of rhombencephalitis

Group B:

- Early autonomic dysregulation without frank cardiopulmonary failure
- Defined as 2 or more of:
    - body temperature 2 or more measurements 4 or more hours apart ≥40°C (rectal, correct for axillary/oral/eardrum)
    - resting tachycardia up to 150/min
    - hypertension: systolic blood pressure >90th percentile for age (using table)
    - hyperglycaemia (without IV dextrose)

Exclusion criteria

- Severe illness meeting the criteria for the primary clinical endpoint below
- HFMD with aseptic meningitis or febrile seizure as only complication

Definitions

HFMD: Febrile illness with papulovesicular rash on palms and soles, with or without vesicles/ulcers in the mouth. Rash may occasionally be maculopapular without vesicular lesion, and may also involve the buttocks, knees or elbows, particularly in younger children and infants (*26*).

Herpangina: Febrile illness with multiple oral ulcers on the posterior parts of the oral cavity.

Aseptic meningitis: Febrile illness with headache, vomiting and meningism associated with presence of more than 5–10 white cells per cubic millimeter in cerebrospinal (CSF) fluid, and negative results on CSF bacterial culture (*26*).

Wandering eyes: Rotary eye movement without fixation (*26*)

Simple febrile seizure: A febrile seizure is a seizure accompanied by fever (temperature >38°C by any method), without central nervous system infection, that occurs in infants and children 6 through 60 months of age. Simple febrile seizures were defined as primary generalized seizures that lasted for less than 15 minutes and did not recur within 24 hours (*52*).

Primary Endpoints

Efficacy

Advanced stage HFMD with frank autonomic dysregulation or cardiopulmonary failure within 48 hours. Defined as one of:

- resting tachycardia >150
- systolic blood pressure >95th percentile for age
- tachypnea
- profuse sweating
- hypotension/shock
- pulmonary edema/hemorrhage
- cardiac arrest

Safety

AE/SAE, particularly known side effects of IVIg or fluid overload

Secondary endpoints

• signs and symptoms after 24h to 48h

• time to resolution of physiologic derangements (tachycardia, tachypnea, fever)

• 7-day mortality

- duration of hospitalization
- need for and duration of mechanical ventilation
- need for rescue therapy (milrinone, CRRT/ECMO, inotropic support, other rescue therapy)
- neurologic sequelae at discharge and 6mo follow-up, including neurodevelopmental assessment using Bayley score
- MRI abnormalities at discharge
- subgroup analysis of patients who did and did not receive phenobarbital before randomization (part of guidelines in Vietnam)
- EV or EV71 qPCR positivity in rectal/NP swabs on day 1-2-3-4-5 or until discharge and after 2 weeks at follow-up (all qPCRs will be performed at a single location)
- acute, before and after study drug dosage 1 and 2 and convalescent serum (after 2 weeks at follow-up)
- CRP and procalcitonin levels at randomization
- cardiac troponin I or T at randomization
- natriuretic peptide β at randomization
- cytokines (G-CSF, IL-5) levels at randomization and after 1st and 2nd study drug
- cost-effectiveness

Subsidiary studies

- subgenotyping, whole genome sequencing of viruses
- qPCR positivity for enteroviruses / EV71 of blood day 1/ CSF (if collected)
- host genetics
- cardiac ultrasound at day 1, 2, 3, discharge
- IVIg neutralization titers of different IVIg brands
